# Endoscopic mucosal resection: still a reliable therapeutic option for gastrointestinal neuroendocrine tumors

**DOI:** 10.1186/s12876-021-01821-6

**Published:** 2021-05-24

**Authors:** Gholam Reza Sivandzadeh, Fardad Ejtehadi, Shima Shoaee, Ladan Aminlari, Ramin Niknam, Ali Reza Taghavi, Bita Geramizadeh, Ahmad Hormati, Ali Reza Safarpour, Kamran Bagheri Lankarani

**Affiliations:** 1 Department of Internal Medicine, Gastroenterohepatology Research Center, School of Medicine, Shiraz University of Medical Sciences, Shiraz, Fars Iran; 2 School of Medicine, Shiraz University of Medical Sciences, Shiraz, Fars Iran; 3 Gastroentrohepatology Research Center, Shiraz University of Medical Sciences, Shiraz, Fars Iran; 4 Department of Pathology, School of Medicine, Shiraz University of Medical Sciences, Shiraz, Fars Iran; 5 Gastrointestinal and Liver Diseases Research Center, Iran University of Medical Sciences, Firozgar Hospital, Tehran, Iran; 6 Gastroenterohepatology Research Center, Shiraz University of Medical Sciences, Shiraz, Iran; 7 Health Policy Research Center, School of Medicine, Institute of Health, Shiraz University of Medical Sciences, Building No. 2, Eighth Floor, Zand Avenue, 71345-1414 Shiraz, Fars Iran

**Keywords:** Endoscopic mucosal resection, Neuroendocrine tumors, Gastrointestinal NET, NET, ESD

## Abstract

**Background:**

Neuroendocrine tumors (NETs), as a rare and heterogeneous category of solid tumors, feature various morphologies and behaviors. In recent years, the incidence of NETs has continued to increase. Endoscopic mucosal resection (EMR) is one of the therapeutic modalities for the treatment of gastric and rectal NETs.

**Methods:**

We evaluated patients with well-differentiated NETs of the stomach, duodenum, or rectum between 2011 and 2018. In this study, all cases with tumors confined to the mucosal or submucosal layers and smaller than 20 mm were resected using the EMR technique. We used EUS, CT scan, or MRI to exclude patients with advanced disease. All patients were actively monitored for recurrence according to the recommended protocols.

**Results:**

A total of 36 patients with NETs entered the study; 17 (47.2%) were female and the remaining 19 (52.8%) were male, with a total age range of 20–74 years (mean: 52.47 ± 13.47 years). Among the tumors, 31 cases (86.1%) were G1 and the remaining 5 (13.9%) were G2. Based on the pathology reports, 22 tumors (61.1%) were smaller than 1 cm, while the remaining 14 (38.9%) were between 1–2 cm. Twenty-two patients (61.1%) had a margin of specimen involved with the tumor. No recurrence was observed during the mean follow-up time of 63.5 ± 19.8 months (range: 39–103 months). All 36 cases survived during the study period.

**Conclusion:**

Conventional EMR procedure provides low chance of R0 (complete resection) achievement in gastrointestinal NETs smaller than 20 mm and limited to the mucosa or sub mucosa. However, it could be an option if patients are closely followed. Postoperative marginal involvement is not a reliable predictor of disease recurrence, which may be explained by the deleterious effect of heat coagulation and cauterization applied during tumor removal.

## Introduction

Gastrointestinal neuroendocrine tumors (NETs), called carcinoid tumors, feature a wide spectrum of malignant potential and originate from enterochromaffin-like (ECL) cells. Gastric NETs involve 7 to 8% of all neuroendocrine tumors and are classified into three main types. The first type accounts for 75% of patients and is associated with chronic atrophic gastritis [1, 2], while the second accounts for 6% of cases and is linked with multiple endocrine neoplasm (MEN) disease (including Zollinger-Ellison syndrome) and the third is the most invasive type with a high chance of metastasis [3, 4].

In recent years, the prevalence of gastrointestinal NETs has increased such that in 2004, the incidence of NETs was 5.25 per 100,000 population, while in 1973, this rate was only 1.09 cases per 100,000 [1–3]. Despite the increase in the prevalence and detection of NETs, significant improvements in overall survival have been observed, which might be due to greater awareness of NETs and improvements in medical imaging and endoscopy [5]. Endoscopic mucosal resection (EMR) and endoscopic sub mucosal dissection (ESD) are the two endoscopic techniques used to treat NETs. The EMR technique is a relatively simple technique requiring unsophisticated skills and inexpensive tools. This technique is used for well-differentiated gastric or duodenal lesions smaller than 20 mm in diameter and well-differentiated tumors confined to the mucosal layer without invasion to the lymph nodes or blood vessels. Similar to the NETs of the upper gastrointestinal tract, those confined to the rectum may be amenable to endoscopic removal. NETs of the rectum that are smaller than 10 mm in diameter are typically confined to the submucosal layer. These small tumors are usually not invasive; therefore, they are good candidates for colonoscopic removal by EMR or ESD. The incomplete removal of the tumoral lesion, as one of the essential causes of recurrence, is among the high risks accompanied by the EMR procedure. Hence, the ESD technique is more favored as it diminishes the chance of incomplete excision. Besides, ESD enables the complete excision of the lesion both vertically and peripherally within an en bloc sample, which permits a more accurate pathological evaluation. Nonetheless, ESD takes a longer amount of time and requires more sophisticated skills and increased resources [6–9].

This study was designed to address the long-term follow-up of cases with localized NETs in either rectum or upper gastrointestinal (GI) tract who were treated by EMR procedure.

## Patients and methods

This study was conducted at Shiraz University of Medical Sciences in accordance with relevant guidelines and regulations. After obtaining study approval from the ethics committee of Shiraz University of Medical Sciences (IR.SUMS.MED.REC.1397.110), all cases with pathological confirmation of NET (either with biopsy or after resection) from October 2011 through March 2018 were entered into the study. Patients with advanced tumors unfit for endoscopic removal (larger than 2 cm in size,, invasion of muscularis properia on endoscopic ultrasound [EUS], and/or poorly differentiated lesions) were excluded, as well as those with non-gastrointestinal involvement, non-endoscopic tumor excision, inability to obtain the desired information, and/or lost on follow-up. Informed consent was obtained from all patients and the option of surgery was made to all cases with margin involvement, particularly to those with NET in the duodenum.

In the beginning, EUS was conducted using a radial array echoendoscope for all patients with gastroduodenal and rectal lesions for precise local staging prior to resection. Then, cases with lesions limited to the mucosa or submucosa and less than 2 cm in diameter were considered for EMR. In this study we did not excluded cases with size up to 2 cm if there was no lymph node metastasis or invasion to muscularis propria (MP) based on pre resection endosonography findings. After conscious sedation under the supervision of an anesthesiologist, EMR was performed with either snare polypectomy after submucosal saline injection or via the band ligation method based on the gastroenterologist’s preference and the size/location of the tumor. In all of patients at the time of endoscopic resection any visualized remnant tissue was cauterized with the tip of snare. A CT scan, MRI, and/or Octreoscan was employed when appropriate to evaluate the disease extent and distant metastasis according to the relevant guidelines [9–12]. After resecting the lesion, follow-up program consisted of endoscopic examinations at three, six, and twelve month intervals. In those with incomplete resection follow-up pursued at six to twelve month intervals, thereafter. Either CT scan or MRI was performed at 12-month intervals. During follow-up endoscopic examinations defoaming agent Simethicone was applied for thorough and clear visualization. NBI was applied in order to increase the yield of study. Biopsy was taken from any suspected lesion. At the time of data collection, all cases were recalled and reevaluation for survival and tumor recurrence was meticulously performed. Tumors were classified according to the 2010 World Health Organization classification based on the assessment of mitotic rate and/or Ki-67 index [13]. All pathologic reports were reviewed and appropriate information including the size of the lesion, grading, and margin clearance (R0) status was recorded.

### Statistical analysis

Statistical Package for the Social Sciences Version 19.0 (SPSS Inc., Chicago, IL, USA) was used to analyze the data. Frequency (%) and mean ± standard deviation were also used as descriptive statistics.

## Results

Forty-four cases with definite pathologic diagnosis of NET were evaluated in this study. Six patients underwent surgical resection due to having tumors with a high grade (G3), unsuitable location (ileum), or advanced stage (> T1). Furthermore, two cases were lost during follow-up. Table 1 demonstrates the demographic data and clinical features of NETs. Ultimately, 36 patients entered the study; seventeen (47.2%) were female and the remaining 19 (52.8%) were male, with, a total age range of 20–74 years (mean: 52.47 ± 13.47 years). Fifteen tumors (41.7%) were located in the stomach, 12 (33.3%) in the duodenum, and 9 (25%) in the rectum. Of these, 31 tumors (86.1%) were G1 and the remaining 5 tumors (13.9%) were G2. Based on the pathology report, 22 tumors (61.1%) were smaller than 1 cm in diameter while 14 (38.9%) were between 1–2 cm. Only 4 patients were treated with modified EMR (i.e. cap or suction assisted EMR) and all of them had no resection margin involvement. All of them were below 1 cm.Twenty-two (61.1%) patients had a margin of specimen involved with the tumor. Out of the tumors with positive margins, 69.6% were smaller than 1 cm and 30.4% were between 1–2 cm. No case with lymphovascular invasion was reported in pathological specimens. Table 2 summarizes the tumor margin involvement status based on different primary tumor locations and sizes. No recurrence was observed during the mean follow-up time of 63.5 ± 19.8 months (range: 39–103 months). The mean follow-up in patients with sample margin involvement was 56.7 ± 17.8 months (range: 39–97 months), compared with 74.2 ± 18.4 months (range: 42–103 months) among patients with sample margin clearance. All 36 cases undergoing EMR survived during the study period. One patient in the surgical group died due to cardiovascular disease and senility.

**Table 1:** Demographic characteristics and clinical features of patients with neuroendocrine tumors (NETs)

Number of patients	36
Male sex, n (%)	19 (52.8%)
Female sex, n (%)	17 (47.2%)
Age, years [mean (range)]	52.47 ± 13.47 (20–74)
Location of tumor	
Stomach	15(41.7%)
Duodenum	12 (33.3%)
Rectum	9 (25%)
WHO tumor grade	
G1	31 (86.1%)
G2	5 (13.9%)
Tumor size	
< 1 cm	22(61.1%)
1–2 cm	14 (38.9%)
Resection margin	
Involved	22(61.1%)
Free	14 (38.9%)
Follow-up, month [mean (range)]	
All patients with EMR	63.5 ± 19.8
Patients with free resection margin	74.2 ± 18.4
Patients with involved resection margin	56.7 ± 17.8

**Table 2:** Tumor margin involvement status based on different tumor primary locations and sizes

Tumor margin involvement	Tumor size	
	< 1 cm	1–2 cm
*Yes (positive margin)*		
Stomach	7	1
Bulb	4	5
Rectum	5	0
*No (clear margin)*		
Stomach	4	3
Bulb	1	2
Rectum	1	3

## Discussion

Neuroendocrine tumors encompass a heterogeneous group of malignancies capable of secreting certain hormones and amines. In western countries, the annual incidence and prevalence of NETs have been calculated as 3.56 per 100,000 and 48 per 100,000 individuals, respectively. In the past several decades, these tumors have been increasingly recognized, which is thought to be due to increased awareness of NETs and the advent of sophisticated radiological modalities. Concurrently, early detection and potent therapeutic options have led to improved survival of patients with NETs. These tumors are classified into well-differentiated or poorly differentiated groups based on pathological morphology, mitotic rate, and the Ki-67 index. Accordingly, tumors are designated as G1, G2, or G3 [12, 14, 15].

Conventional polypectomy, EMR, and ESD are considered as therapeutic endoscopic modalities for treating gastric, duodenal, and rectal NETs with limited penetration into the submucosa [15]. Conventional polypectomy using a snare is a simple procedure. Although few studies showed that polypectomy might yield a high degree of free resection margins in small and superficial NETs, most of these tumors have already penetrated the submucosa, which may lead to decreased curability of conventional polypectomy [16, 17]. EMR can be performed with or without a cap or ligation device. It is a relatively simple procedure with a rapid learning curve and much less utilization of resources than ESD. EMR may achieve complete pathological resection in 52% to 84.6% of NETs [18, 19]. On the other hand, ESD requires sophisticated equipment and increased expertise. The major advantage of ESD is its capability to achieve a better lateral and vertical resection margin at the expense of a much greater risk of bleeding or perforation [20]. Sato et al. showed in a low number of cases that about 66% of NETs resected by EMR had a positive vertical margin, whereas no case with ESD had positive vertical or horizontal margins [21].

In this study, we performed EMR for the resection of all GI NETs even larger than 1 cm ( up to maximum sizes of 2 cm in diameter) if there was no evidence of invasion to muscularis propria (MP) and no lymph node enlargement based on pre resection endosonographic findings. The management of intermediate-sized NETs (between 1-2 cm) confined to mucosa or submucosa is somewhat controversial. Other than tumor size and depth, additional factors including mitotic index and lymphovascular invasion could notoriously affect patients prognosis [22–24]. Some previous studies have shown that gastric NET up to 2 cm are amenable to endoscopic resection [1, 25–27]. While involvement of MP should be considered a contraindication for local resection at least with EMR, the size up to 2 cm may still response to local resection. In our study, out of 14 (38.9%) cases who had NETs between 1-2 cm, six (42.8%) patients did not achieved complete resection after EMR. However, none of them experienced a recurrence. As a result, our findings show that EMR could be conducted in NET between 1–2 cm if there is no lymphovascular invasion or involvement of MP. In our study, the majority of tumors were G1 and smaller than 1 cm. However, despite the reports of similar studies, about 61% of our cases failed to achieve a clear resection margin after EMR. Out of 22 tumors with incomplete resection, 14 (63.6%) were located in the duodenum and rectum. Out of 16 tumors less than 1 cm in size and positive margin after resection, 56.2% were located in the duodenum and rectum. Presumably, both difficulty in EMR within the duodenum and more conservative approaches implemented by physicians to avoid complications during EMR in the rectum and duodenum are factors leading to the higher number of tumors with incomplete resection despite smaller sizes. Several studies on rectal NETs showed that the complete resection rate was significantly higher after ESD relative to simple EMR but was comparable to modified EMR (EMR with banding or using a cap and suction) [19, 28]. Surprisingly, other studies, including a meta-analysis on 14 studies and 823 cases, concluded that modified EMR with the suction method (93.65%) is superior to ESD (84.08%) in achieving free resection margins in rectal NETs smaller than 10 mm [29–31]. The modified EMR (m-EMR) technique using either suction cap or ligation band is much more effective in achieving complete resection and R0 margin comparing to conventional EMR procedure which is performed by a snare after submucosal saline injection [19]. In our study, only 4 patients were treated with m-EMR procedure. All of these cases achieved complete resection (R0). On the other hand, conventional EMR procedure was implemented in the remaining 32 cases. As a result of high number of conventional EMR procedures done we could only achieve a 40% complete resection rate based on histology.

In this study, cases with positive resection margins were meticulously followed according to recommended guidelines and no further resections were undertaken. We followed all cases with either border involvement or free resection margins for approximately 5 years. On follow-up, no case with tumor recurrence was detected despite a high margin involvement rate among our patients. This is in concordance with the series reported by Weili Sun et al., who achieved complete resection in 31 out of 41 (75.6%) with EMR and 30 out of 35 (85.7%) cases with ESD. Interestingly, in those 14 cases with positive resection margins, they merely followed the patients and no recurrence was reported [20]. It is presumed that the electrocauterization effect with heat generation may destruct the remnant of malignant cells and preclude tumor relapse despite margin involvement in the pathological specimen. Furthermore, in our study, most of the resected tumors with border involvement after resection were smaller than 10 mm, increasing the chance of tumor cell degeneration with electrocauterization.

In this series, there was no instance of perforation or major bleeding. However, this might be due to the characteristics of the patients and expertise of the performers, so it could not be generalized.

The strong point of this study was its long-lasting follow-up of patients with an acceptable number of cases compared to similar small studies. However, despite the remarkable results, this study still has several limitations. First, this work was a retrospective study that may be associated with the risk of selection bias. Furthermore, this study is categorized as a single-center case series without comparison with ESD or modified EMR.

In conclusion, conventional EMR procedure provides low chance of R0 (complete resection) achievement in gastrointestinal NETs smaller than 20 mm and limited to the mucosa or sub mucosa, but still EMR could be an option in low grade lesions up to 20 mm without lymph node metastasis or invasion to muscularis propria if patients are closely followed. Whenever EMR is performed, particularly where and when equipment, instruments, or adequate expertise for ESD are not readily accessible, incomplete resection of the lesion is not relevant for the prognosis of these patients and tumor recurrence, especially when cauterization of the margins of the lesion is done to ablated the residual tumor. This latter result is very interesting and might be topic for a different study.

## Data Availability

All data and materials are available from the first author and corresponding author.
